# Mitochondrial double-stranded RNA triggers induction of the antiviral DNA deaminase APOBEC3A and nuclear DNA damage

**DOI:** 10.1016/j.jbc.2023.105073

**Published:** 2023-07-19

**Authors:** Chloe Wick, Seyed Arad Moghadasi, Jordan T. Becker, Elisa Fanunza, Sunwoo Oh, Elodie Bournique, Rémi Buisson, Reuben S. Harris

**Affiliations:** 1 Department of Biochemistry, Molecular Biology and Biophysics, University of Minnesota, Minneapolis, Minnesota, USA; 2 Department of Biochemistry and Structural Biology, University of Texas Health San Antonio, San Antonio, Texas, USA; 3 Department of Life and Environmental Sciences, University of Cagliari, Cittadella Universitaria di Monserrato, Monserrato, Cagliari, Italy; 4 Department of Biological Chemistry, School of Medicine, University of California Irvine, Irvine, California, USA; 5 Center for Epigenetics and Metabolism, Chao Family Comprehensive Cancer Center, University of California Irvine, Irvine, California, USA; 6 Howard Hughes Medical Institute, University of Texas Health San Antonio, San Antonio, Texas, USA

**Keywords:** APOBEC3A, cancer mutagenesis, DNA damage response, innate immune signaling, mitochondrial dsRNA

## Abstract

APOBEC3A is an antiviral DNA deaminase often induced by virus infection. APOBEC3A is also a source of cancer mutation in viral and nonviral tumor types. It is therefore critical to identify factors responsible for APOBEC3A upregulation. Here, we test the hypothesis that leaked mitochondrial (mt) double-stranded (ds)RNA is recognized as foreign nucleic acid, which triggers innate immune signaling, APOBEC3A upregulation, and DNA damage. Knockdown of an enzyme responsible for degrading mtdsRNA, the exoribonuclease polynucleotide phosphorylase, results in mtdsRNA leakage into the cytosol and induction of APOBEC3A expression. APOBEC3A upregulation by cytoplasmic mtdsRNA requires RIG-I, MAVS, and STAT2 and is likely part of a broader type I interferon response. Importantly, although mtdsRNA-induced APOBEC3A appears cytoplasmic by subcellular fractionation experiments, its induction triggers an overt DNA damage response characterized by elevated nuclear γ-H2AX staining. Thus, mtdsRNA dysregulation may induce APOBEC3A and contribute to observed genomic instability and mutation signatures in cancer.

The apolipoprotein B mRNA editing catalytic polypeptide-like 3 (APOBEC3 or A3) family of proteins comprises seven members in humans (1). As single-stranded (ss)DNA cytosine deaminases, these enzymes normally function as antiviral factors capable of inhibiting virus replication, suppressing infectivity, and blocking pathogenesis (2). However, this potent DNA editing activity can also be directed at the human genome in cancer and cause mutations in chromosomal DNA (3, 4, 5). A3-catalyzed genomic C-to-U deamination events become immortalized as C-to-T transition and C-to-G transversion mutations, most frequently in TCA and TCT trinucleotide motifs. Collectively, these single base substitution (SBS) mutation patterns in cancer are known as SBS2 and SBS13 or, more simply, as the “APOBEC mutation signature.” The APOBEC mutation signature is found in over 70% of cancers and can be the largest fraction of somatic variation in many individual tumors and tumor types (6, 7).

APOBEC3A (A3A) and APOBEC3B (A3B) are the most likely sources of APOBEC signature mutations in cancer (most recently addressed by (8, 9)). Both enzymes are potent ssDNA cytosine deaminases that intrinsically prefer TC motifs due to identical loop regions that engage the thymine nucleobase immediately upstream of a target cytosine (10, 11). Ectopic expression of both enzymes inflicts APOBEC signature mutations in model bacteria and yeast systems, the chicken cell line DT40, and the human cell line HAP1 (3, 9, 12, 13, 14, 15, 16). Recently, CRISPR knockout studies have shown that both enzymes contribute to ongoing mutagenesis in human cancer cell lines, with A3A accounting for a larger fraction of the overall APOBEC signature (8). Importantly, each of these human enzymes is capable of catalyzing mutagenesis and promoting tumor formation in mice, which demonstrates that this mutational process is capable of uniquely driving carcinogenesis (and is not simply a passenger phenomenon despite the fact that most APOBEC signature mutations are likely to be aphenotypic) (17, 18, 19, 20, 21).

*A3A* expression is suppressed in most normal human tissues (22, 23, 24). However, consistent with its function as an antiviral innate immune factor, its transcription can be induced by viral infection (25, 26, 27). For instance, human papillomavirus infection of normal immortalized keratinocytes or human tonsillar epithelial cells, human polyomavirus infection of human urothelium, and human cytomegalovirus infection of decidual tissues are all reported to trigger increased expression of *A3A* (26, 28, 29, 30). Furthermore, consistent with antiviral function, A3A is induced by type I interferons (IFNs) in multiple cell types including monocytes, macrophages, and dendritic cells (22, 31, 32, 33, 34). This pathway is initiated by IFN binding to its cell surface receptor, JAK/STAT signal transduction, and STAT2 binding the *A3A* promoter and transcriptional activation (25). However, it is important to note that infection by other viruses such as the lentivirus HIV-1 and the herpesvirus Epstein–Barr virus fails to induce *A3A* expression (35, 36). Moreover, most cancer types with an APOBEC signature SBS2 and SBS13 and *A3A* expression lack viral etiologies (7, 23, 24, 37, 38, 39). It is therefore of considerable interest to understand nonviral mechanisms of *A3A* upregulation.

Extrinsic nucleic acids from dead cells and intrinsic nucleic acids from chromosome missegregation (micronuclei) and aberrant endogenous virus and transposon activity can, like viral nucleic acids, activate nucleic acid sensors and trigger strong IFN responses including *A3A* upregulation (31, 40, 41, 42). Mitochondria are another potential source of endogenous immunostimulatory nucleic acids (43, 44, 45). For instance, bidirectional transcription of mitochondrial genes can result in double-stranded (ds)RNA, which is normally recycled by a degradosome comprising the exoribonuclease polynucleotide phosphorylase (PNPase) and the ATP-dependent RNA helicase SUPV3L1 (46, 47, 48, 49, 50). Knockdown of either component of this complex results in accumulation of mitochondrial dsRNA (mtdsRNA) (45, 51). Moreover, PNPase depletion additionally allows mtdsRNA to escape into the cytosol (45, 52). Cytosolic mtdsRNA is then free to engage the RNA sensors RIG-I and MDA5 and potentiate an IFN response (45). Therefore, a combination of genetic, biochemistry, and cell biology approaches is used here to test the hypothesis that mtdsRNA can be mistaken as foreign and trigger a virus-like innate immune response that leads to *A3A* induction and nuclear DNA damage.

## Results

### Mitochondrial and nuclear dsRNA trigger A3A upregulation

**Figure 1:**
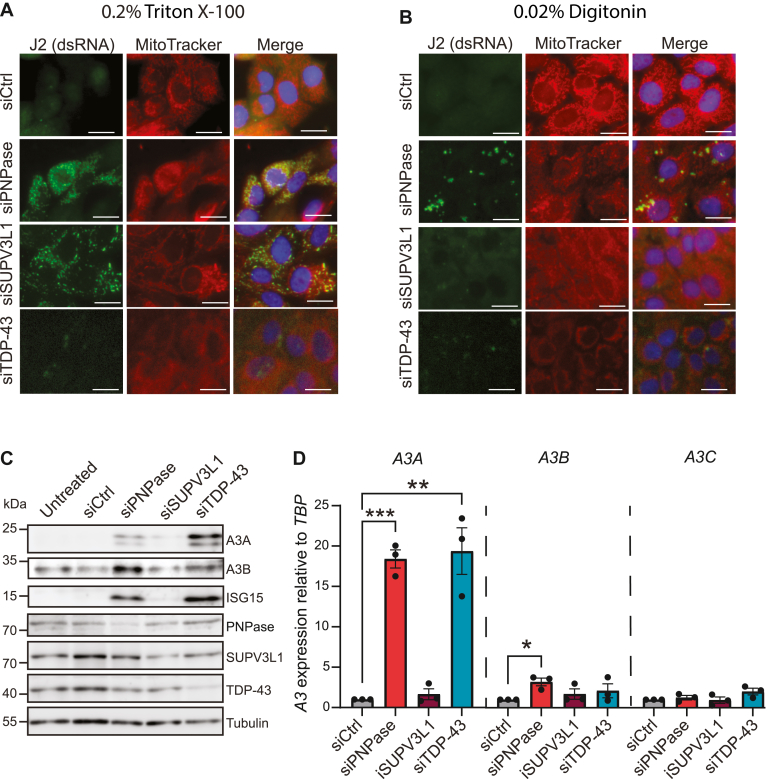
**Leaked mitochondrial dsRNA triggers A3A upregulation**. *A* and *B*, immunofluorescence microscopy images of MCF10A cells treated with siCtrl, siPNPase, siSUPV3L1, or siTDP-43 for 72 h and permeabilized with (*A*) 0.2% Triton X-100 or (*B*) 0.02% digitonin after which they were stained with the dsRNA-binding antibody J2. Mitochondria were stained with MitoTracker, and nuclei were stained with Hoechst (the scale bar represents 10 μm). *C*, immunoblot analysis of the indicated proteins expressed in MCF10A cells treated with siCtrl, siPNPase, siSUPV3L1, or siTDP-43 for 72 h. Tubulin was used as a loading control. All subpanels are from the same representative blot. *D*, Reverse transcription-quantitative PCR analysis of *A3* mRNA levels in MCF10A cells after treatment with siCtrl, siPNPase, siSUPV3L1, or siTDP-43 for 72 h. Expression refers to *A3* mRNA fold change relative to the negative control (set to 1) normalized to *TBP*. Mean values ± SEM of three independent experiments (∗*p* ≤ 0.05, ∗∗*p* ≤ 0.01, ∗∗∗*p* ≤ 0.001 by Student’s *t* test and not shown if insignificant).

To test the hypothesis that mtdsRNA leads to an induction of *A3A* expression, the breast epithelial cell line MCF10A was transfected with siRNAs to deplete the mitochondrial exoribonuclease PNPase and the RNA helicase SUPV3L1 and immunofluorescent microscopy was used to quantify dsRNA. Strong cytoplasmic staining with the dsRNA-specific monoclonal antibody J2 was observed in PNPase- and SUPV3L1-depleted cells after membrane permeabilization with 0.2% triton-X100 (45) (Fig. 1*A*). A stringent 0.2% digitonin permeabilization protocol yielded similar results (Fig. S1*A*). The majority of the dsRNA signal in these conditions appeared coincident with mitochondria as indicated by overlapping staining with MitoTracker (Red CMXRos). Interestingly, a milder 0.02% digitonin protocol, which permeabilizes only the plasma membrane (and not mitochondrial or nuclear membranes (53)), indicated that only PNPase depletion selectively triggers cytosolic dsRNA accumulation (Fig. 1*B*; additional images in Fig. S1*B*). In comparison, when using the same 0.02% digitonin treatment to preferentially permeabilize the cytoplasmic membrane, SUPV3L1 depletion did not lead to significant mtdsRNA leakage into the cytosol (Fig. 1*B*; additional images in Fig. S1*B*). As a negative control, non-digitonin-permeabilized cells showed little J2 staining (images in Fig. S1*C*). Quantification of imaging results from the 0.2% and 0.02% digitonin experiments confirmed significant overlap between dsRNA and MitoTracker staining (Fig. S1*D*) and significant numbers of dsRNA foci accumulating in PNPase-depleted cells (Fig. S1*E*).

To assess if knockdown of PNPase and subsequent release of dsRNA into the cytosol causes an IFN response in MCF10A cells, the expression of the interferon-stimulated gene *ISG15* was measured as an indicator of a type I IFN production. PNPase knockdown, but not SUPV3L1 knockdown, resulted in strong upregulation of both ISG15 and A3A (Fig. 1, *C* and *D*). The two isoforms of A3A beginning at Met1 and Met13 are both evident, consistent with a transcriptional induction mechanism. Indeed, *A3A* mRNA levels increased 15- to 20-fold through PNPase knockdown in comparison with a nontargeting siRNA (Fig. 1*D*). *A3B* mRNA levels were also induced significantly, but other *A3* mRNAs appeared unchanged (Fig. 1*D*; quantification of all *A3* mRNAs in Fig. S2*A*). Similar results for A3A and A3B were obtained in the lung carcinoma epithelial cell line A549 but not in HeLa cells, which are defective in interferon synthesis (Fig. S2, *B* and *C*). Taken together, these data indicated that leakage of mitochondrial dsRNA into the cytosol leads to a strong upregulation of A3A and a weaker but still significant induction of A3B.

To determine if dsRNA of a nonmitochondrial origin might also lead to *A3A* induction, the RNA regulatory protein TAR DNA-binding protein 43 (TDP-43) was knocked down, which is known to result in cytoplasmic RNA polymerase III transcript accumulation (54, 55). Thus, TDP-43 was depleted from MCF10A cells and, as anticipated from this prior literature, this knockdown caused an accumulation of dsRNA puncta in the cytoplasm (Fig. 1, *A* and *B*). Importantly, this dsRNA signal showed little overlap with mitochondrial staining by MitoTracker Red CMXRos. However, similar to depletion of PNPase above, immunoblot and reverse transcription-quantitative PCR (RT-qPCR) experiments showed a >10-fold increase in A3A levels following TDP-43 depletion (Fig. 1, *C* and *D*). It is not clear why TDP-43 depletion results in higher A3A protein levels in comparison with PNPase depletion, despite similar fold-induction at the mRNA level and similarly high IFN responses as assessed by ISG15 levels. Nevertheless, despite this additional protein-level curiosity, these results combined to demonstrate that an accumulation of cytosolic dsRNA from mitochondrial or nuclear origins leads to a robust induction of A3A expression.

### Cytosolic sensing of mitochondrial dsRNA requires the RNA sensor RIG-I

**Figure 2:**
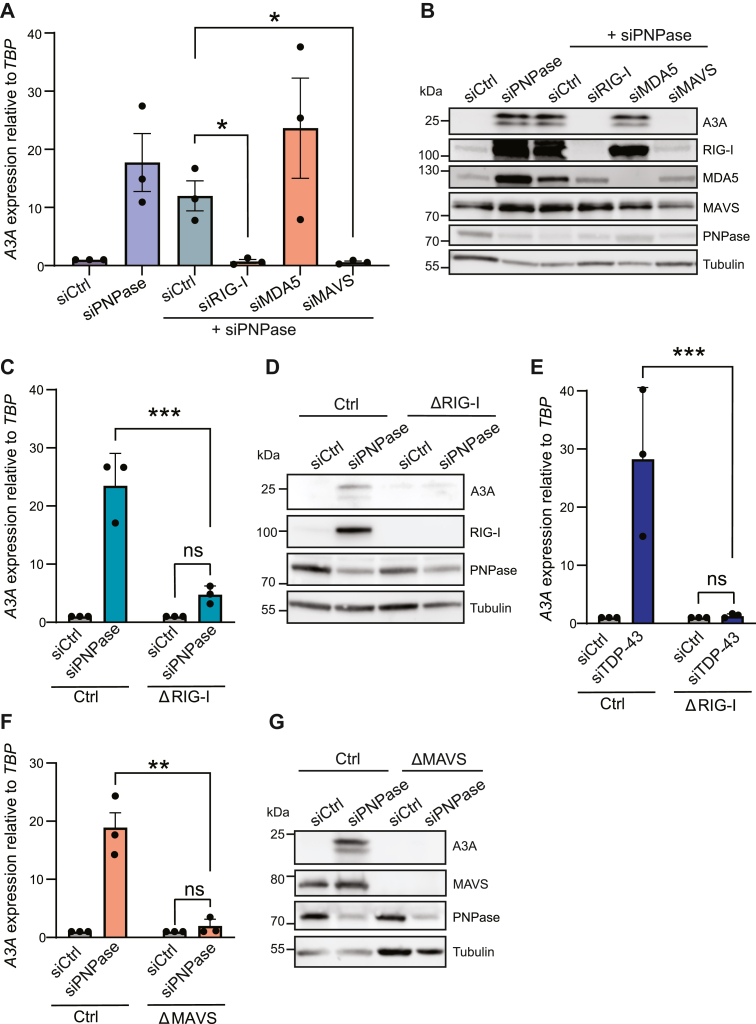
**The RIG-I/MAVS axis is required for upregulating A3A in response to endogenous mitochondrial dsRNA**. *A*, RT-qPCR analysis of *A3A* after treatment with siCtrl, siPNPase, and codepletions of siPNPase with siCtrl, siRIG-I, siMDA5, and siMAVS for 72 h in MCF10A cells. Expression refers to mRNA fold change relative to the negative control (which was set to 1) and was normalized to *TBP*. Mean values ± SEM of three independent experiments (∗*p* ≤ 0.05 by Student’s *t* test and not shown if insignificant). *B*, immunoblot analysis of A3A in MCF10A cells treated with siCtrl, siPNPase, and codepletions of siPNPase with siCtrl, siRIG-I, siMDA5, and siMAVS. Tubulin was used as a loading control. *C* and *D*, RT-qPCR and immunoblot analysis of A3A mRNA and protein levels, respectively, in control or *RIG-I* KO MCF10A cells following siCtrl or siPNPase treatment. Expression refers to mRNA fold change relative to the negative control (which was set to 1) and was normalized to *TBP*. Mean values ± SEM of three independent experiments (∗∗∗*p* ≤ 0.001 by Student’s *t* test and not shown if insignificant). *E*, RT-qPCR analysis of *A3A* mRNA levels, respectively, in control or *RIG-I* KO MCF10A cells following siCtrl or siTDP-43 treatment. Expression refers to mRNA fold change relative to the negative control (which was set to 1) and was normalized to *TBP*. Mean values ± SEM of three independent experiments (∗∗∗*p* ≤ 0.001 by Student’s *t* test and not shown if insignificant). *F* and *G*, RT-qPCR and immunoblot analysis of A3A mRNA and protein levels, respectively, in control or *MAVS* KO MCF10A cells following siCtrl or siPNPase treatment. Expression refers to mRNA fold change relative to the negative control (which was set to 1) and was normalized to *TBP*. Mean values ± SEM of three independent experiments (∗∗*p* ≤ 0.01 by Student’s *t* test and not shown if insignificant). RT-qPCR, reverse transcription-quantitative PCR.

To determine the RNA sensor responsible for A3A upregulation in response to mtdsRNA accumulation in the cytoplasm, MCF10A cells were codepleted of PNPase and candidate RNA sensors and then A3A levels were quantified as above. In comparison with the induction of A3A observed in cells depleted for PNPase, codepletion of PNPase and the cytosolic RNA sensor RIG-I prevented *A3A* upregulation. In contrast, treatment with siRNAs against MDA5 had no significant effect (Fig. 2, *A* and *B*). To further substantiate these knockdown results, MCF10A cells engineered by CRISPR-Cas9 to lack RIG-I also demonstrated that this sensor is required for A3A induction by cytoplasmic dsRNA (Fig. 2, *C* and *D*). As anticipated from prior work (55), RIG-I null MCF10A cells also failed to induce A3A following TDP-43 depletion (Fig. 2*E*).

MAVS is an adaptor in RNA sensing that typically functions downstream of RIG-I (56, 57). To further test whether A3A induction is dependent on the sensing of dsRNA, RNAi experiments were done to investigate the involvement of MAVS. As above for RIG-I experiments, codepletion of PNPase and MAVS reduced *A3A* expression to uninduced levels and MAVS-null clones showed a complete abrogation of *A3A* induction following knockdown of PNPase (Fig. 2, *A*, *B*, *F* and *G*). These results combined to further demonstrate that *A3A* is upregulated through the sensing of cytosolic mtdsRNA by the RIG-I/MAVS pathway.

### A3A upregulation by cytosolic mtdsRNA requires STAT2

**Figure 3:**
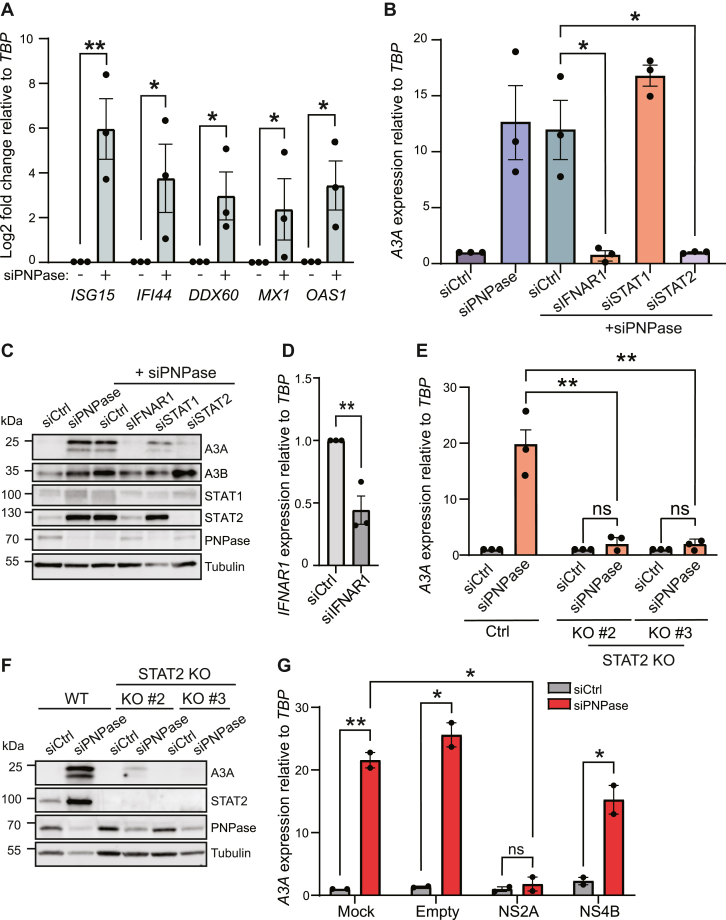
**A3A is upregulated *via* a STAT2-dependent interferon response**. *A*, RT-qPCR analysis of a panel of interferon-responsive genes (*ISG15, IFI44, DDX60, MX1, OAS1*) after siPNPase treatment of MCF10A cells. Expression refers to mRNA log_2_ fold change relative to the negative control (which was set to 0) and was normalized to *TBP*. Mean values ± SEM of three independent experiments (∗*p* ≤ 0.05, ∗∗*p* ≤ 0.01 by Student’s *t* test and not shown if insignificant). *B*, RT-qPCR analysis of *A3A* in MCF10A cells treated with siCtrl, siPNPase, and siPNPase in combination with siCtrl, siIFNAR1, siSTAT1, and siSTAT2 (∗*p* ≤ 0.05 by Student’s *t* test and not shown if insignificant). *C*, immunoblot analysis of MCF10A cells treated with siCtrl, siPNPase, and codepletions of siPNPase in combination with siIFNAR1, siSTAT1, and siSTAT2. *D*, RT-qPCR analysis of *IFNAR1* in MCF10A cells treated with siCtrl or siIFNAR1 and siPNPase. Mean values ± SEM of three independent experiments (∗∗*p* ≤ 0.01 by Student’s *t* test). *E* and *F*, RT-qPCR and immunoblot analysis of *A3A* mRNA and protein levels, respectively, in control or *STAT2* KO MCF10A cells following siCtrl or siPNPase treatment. Expression refers to mRNA fold change relative to the negative control (which was set to 1) and was normalized to *TBP*. Mean values ± SEM of three independent experiments (∗∗*p* ≤ 0.01 by Student’s *t* test and not shown if insignificant). *G*, RT-qPCR analysis of *A3A* in A549 cells treated with siCtrl/siPNPase and transfected with plasmids encoding NS2A, NS4B, or GFP. Expression refers to mRNA fold change relative to the negative control (which was set to 1) and was normalized to *TBP*. Mean values ± SEM of two independent experiments (∗*p* ≤ 0.05, ∗∗*p* ≤ 0.01 by Student’s *t* test and not shown if insignificant). RT-qPCR, reverse transcription-quantitative PCR.

Accumulation of immunostimulatory dsRNA can trigger a wide array of cellular responses, and therefore a set of IFN-responsive genes was analyzed to determine whether the canonical IFN pathway is involved. We found that five canonical IFN-stimulated genes (*ISG15*, *IFI44*, *DDX60*, *MX1*, and *OAS1* (58)) were all significantly upregulated following siPNPase treatment (Fig. 3*A*). Genes encoding the inflammatory cytokines tumor necrosis factor alpha and interleukin 6 were also induced by PNPase knockdown (Fig. S4). The expression of specific *IFN* genes was not examined, but several are also *bona fide* ISGs and induction is anticipated based on prior reports (*e.g*., IFN-β in ref. (45)). To confirm that A3A is upregulated through a type I IFN response, knockdown of the IFN-α/β receptor in siPNPase-treated cells effectively reduced *A3A* expression levels to those of the control (Fig. 3, *B* and *C*). *IFNAR1* depletion was confirmed by RT-qPCR in these experiments because available commercial antibodies did not work in our hands (Fig. 3*D*). These results indicated that, following activation of RIG-I and MAVS, A3A induction occurs through a type-I IFN-dependent signaling pathway.

The most likely mediators of a type-I IFN-dependent response based on the prior literature (25, 59, 60) are the IFN-inducible transcription factors STAT1 and STAT2. These factors were therefore depleted from MCF10A cells with siRNA, and the effect of PNPase knockdown was examined as described above. Interestingly, only *STAT2* (and not *STAT1*) depletion was able to block A3A induction by PNPase knockdown (Fig. 3, *B* and *C*; independent *STAT1* knockdown results in Fig. S3). This important result was confirmed using STAT2-knockout MCF10A clones, where A3A is no longer inducible by PNPase knockdown (Fig. 3, *E* and *F*).

We also extended these results to another cell line using a completely orthologous approach. A549 cells were transfected with vectors expressing the Zika virus proteins NS2A and NS4B as tools to block the JAK-STAT signaling cascade that occurs following IFN induction. NS2A mediates the degradation of STAT1 and STAT2, and NS4B suppresses the phosphorylation of STAT1 (61, 62). A549 cells were transfected concurrently with siPNPase and plasmids encoding FLAG-tagged NS2A and NS4B (Fig. 3*G*). *A3A* upregulation was eliminated by the addition of NS2A. In contrast, transfection of NS4B into the cells had little effect with *A3A* levels still rising 15- to 20-fold after PNPase knockdown. As NS2A (but not NS4B) interferes with STAT2 activation, these data support the knockdown and knockout results above showing that *A3A* induction requires STAT2. Thus, activation of RIG-I/MAVS by endogenous dsRNA causes a type I IFN response that induces *A3A via* STAT2.

### A3A induction by mtdsRNA triggers a DNA damage response

**Figure 4:**
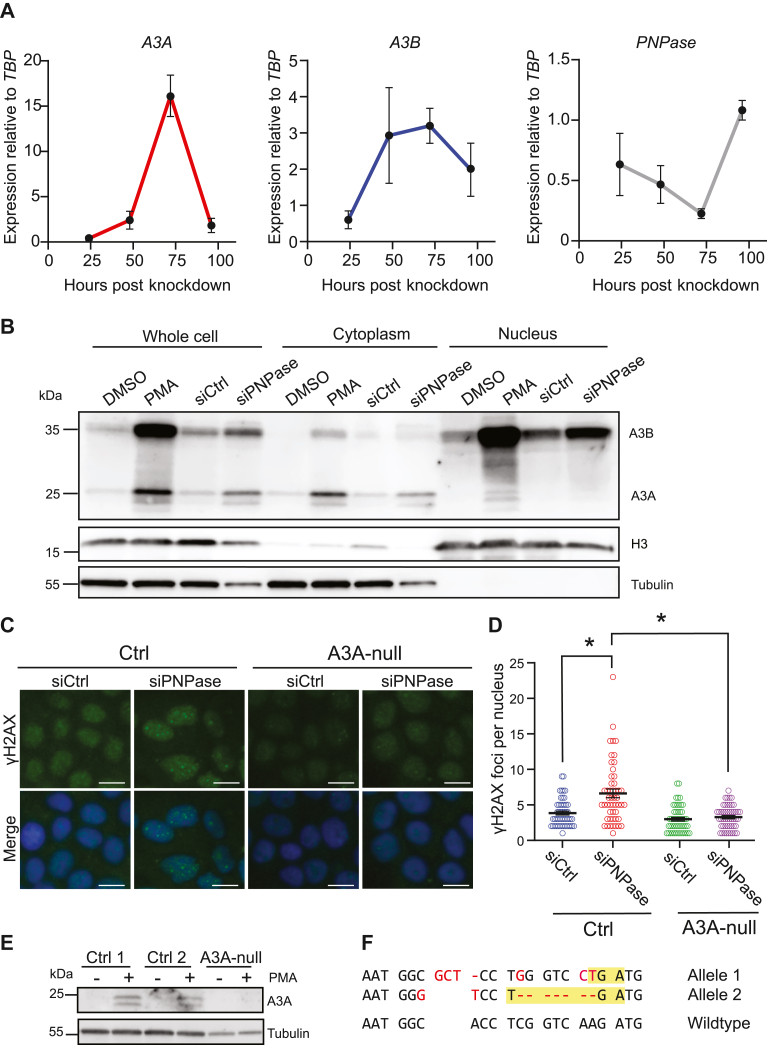
**DNA damage induced by the upregulation of PNPase is A3A dependent**. *A*, Reverse transcription-quantitative PCR analysis of *A3A*, *A3B*, and *PNPase* in MCF10A cells treated with siCtrl/siPNPase after 24, 48, 72, and 96 h (mean ± SEM of three independent experiments). *B*, immunoblot analysis of whole cell, cytoplasmic, and nuclear fractions of MCF10A cells treated with 10 nM siCtrl/siPNPase for 72 h or DMSO/25 ng/ml PMA for 24 h (*C* and *D*) representative immunofluorescence microscopy images of γ-H2AX in control or *A3A* knockout cells treated with siCtrl/siPNPase with quantification of the number of γ-H2AX foci per nucleus (the scale bar represents 10 μm; mean ± SEM of n > 50 cells per condition; ∗*p* ≤ 0.05 by Student’s *t* test and not shown if insignificant). *E*, immunoblot of A3A in two control (*lacZ*) clones and in an *A3A* knockout clone following 24 h of stimulation with 25 ng/ml PMA. *F*, DNA sequence of the CRISPR-disrupted *A3A* alleles in MCF10A cells (5/10 sequenced plasmids had allele 1 and 5/10 allele 2). Frameshift-induced premature stop codons are highlighted in *yellow*, and insertions, deletions, and substitutions are shown in *red*. DMSO, dimethyl sulfoxide; PMA, phorbol 12-myristate 13-acetate.

To investigate the kinetics of A3A induction by mtdsRNA leakage, *A3A*, *A3B*, and *PNPase* mRNA expression levels were analyzed every 24 h over a 4-day period following PNPase depletion (Fig. 4*A*). This analysis revealed that *A3A* expression peaks, approximately 15-fold, at around 72 h after siRNA transfection and quickly recovers to 2-fold induction by 96 h. *A3B* mRNA levels peak with similar kinetics, although only around 3-fold, roughly plateauing between 48 and 72 h post transfection, and *A3B* mRNA levels may also persist slightly longer. In the same time course, *PNPase* mRNA levels are depleted maximally by 72 h post transfection and begin to recover by 96 h (Fig. 4*A*). These results indicate that *A3A* (and *A3B*) mRNA levels correlate inversely with *PNPase* levels (and thereby also with cytosolic mtdsRNA levels) and are likely to be transient in nature.

To determine where mtdsRNA-induced A3A protein accumulates within cells, PNPase was depleted from MCF10A, subcellular fractionation was used to separate nuclear and cytoplasmic components, and immunoblots were done to detect relevant proteins. Phorbol 12-myristate 13-acetate was used as a positive control to induce A3A and A3B, as shown previously (63, 64, 65). This biochemical approach showed that the majority of mtdsRNA-inducible A3A is localized to the cytoplasm, with tubulin as a positive control (Fig. 4*B*). In comparison, the majority of A3B localizes to nuclear fractions, with histone H3 as a positive control (Fig. 4*B*). Cytosolic localization of IFNα-induced endogenous A3A has been reported for another cell line (THP1), and nuclear localization of endogenous A3B has been reported for MCF10A and a multitude of cell lines by many groups (63, 66, 67, 68).

Last, we asked whether the A3A protein induced under these conditions of PNPase depletion/cytosolic mtdsRNA accumulation is capable of inflicting nuclear DNA damage. This was done by depleting PNPase as above from MCF10A cells and then using immunofluorescence microscopy to visualize and quantify the DNA damage marker γ-H2AX. Interestingly, PNPase depletion causes strong increases in both pan-nuclear and focused γ-H2AX staining including a doubling of the number of γ-H2AX foci (Fig. 4, *C* and *D*; quantification in Fig. S5). An independent experiment with doxorubicin as a positive control confirmed this result and suggested that the overall level of DNA damage inflicted by A3A is less than that caused by this chemotherapeutic (Fig. S5). Importantly, MCF10A cells engineered by CRISPR to lack endogenous A3A demonstrated that the majority of these nuclear γ-H2AX foci are dependent upon this enzyme (Fig. 4, *C* and *D*), despite the majority of protein localizing to the cytosol as described above. *A3A* knockout was confirmed by immunoblot and by sequencing the gRNA-binding site where each allele has multiple mutations including a frameshift mutation (Fig. 4, *E* and *F*). These experiments combined to indicate that cytosolic mtdsRNA accumulation leads to a strong A3A-dependent DNA damage response.

## Discussion

**Figure 5:**
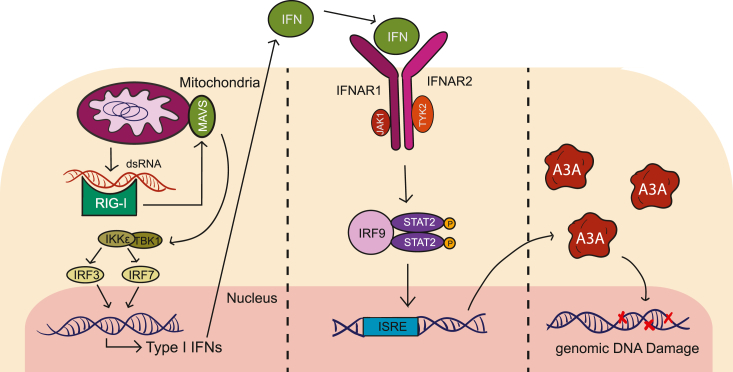
**Working model for A3A upregulation by endogenous dsRNA.** Mitochondrial dsRNAs that accumulate inappropriately in the cytosol are sensed by RIG-I, which signals through the adaptor protein MAVS and leads to a type I interferon response, induction of A3A by STAT2, and chromosomal DNA damage. Three distinct panels are shown to illustrate the fact that interferon signaling can act in both *cis* and *trans* (autocrine and paracrine).

Here, we report that the cytoplasmic accumulation of endogenous dsRNA of mitochondrial origin triggers a strong increase in the expression of A3A and a slight increase in the expression of A3B. While it has been previously reported that foreign and synthetic nucleic acids are able to trigger the induction of A3A through a type-I IFN response (22, 25, 31, 33, 69, 70), our results are the first to examine how dysregulation of endogenous dsRNA may act as a natural source of immunostimulatory nucleic acids and lead to strong upregulation of A3A. We show that the upregulation of A3A by endogenous dsRNA is dependent on the RIG-I/MAVS signaling axis and proceeds through a type I IFN response in a STAT2-dependent manner. Moreover, upregulated A3A, although almost entirely cytoplasmic, is also able to cause chromosomal DNA damage as evidenced by elevated γ-H2AX staining. Taken together, these results support a model in which a breach in mitochondrial integrity can leak dsRNA into the cytosol, which triggers RIG-I/MAVS/STAT2-dependent upregulation of the IFN response including A3A expression and, importantly, DNA damage (Fig. 5). This pathway could be directly relevant to cells with mitochondrial dsRNA leakage as well as to bystander cells due to the auto/paracrine nature of the IFN response. These observations may help explain the periodic (also called episodic) occurrence of APOBEC3 signature mutations in cancer cell lines, which were shown recently to involve A3A (8, 71).

The chromosomal DNA damage observed following knockdown of PNPase and accumulation of mtdsRNA is surprising given that the bulk of induced A3A protein is cytoplasmic. In fact, our subcellular fractionation experiments indicate no detectable A3A in the nucleus of PNPase-depleted cells. This observation is consistent with a prior report of endogenous A3A localization to the cytoplasm following IFN-α treatment of the cell line THP1 (67). However, given the strong genetic dependence of γ-H2AX accumulation here on A3A following PNPase knockdown and cytoplasmic mtdsRNA accumulation, we hypothesize that a low level of induced A3A is able to diffuse through nuclear pores (due to its small size), deaminate single-stranded regions of chromosomal DNA, and trigger DNA breaks as evidenced by elevated levels of nuclear γ-H2AX foci.

In addition to the robust A3A upregulation observed upon knockdown of PNPase, A3B was also significantly induced, although to a much lower extent. A3B has also been found to deaminate genomic DNA, and it is also a major source of mutations in cancer and is found at much higher levels in the nuclear compartment of a wide range of tumors and cancer cell lines (22, 23, 24, 63, 66). Although the majority of DNA damage observed here following PNPase depletion is dependent on A3A, A3B is predominantly nuclear with direct access to chromosomal DNA and, thus, also able to contribute to the overall landscape of APOBEC signature mutations observed in cancer.

Here, we propose that sporadic induction of A3A caused by mitochondrial stress and cytoplasmic dsRNA accumulation over the course of human lifetime may contribute to the overall burden of DNA damage and mutation accumulation in cancer. To investigate the volatility of A3A upregulation due to mitochondrial dysfunction, the kinetics of A3A induction following the depletion of PNPase were investigated. The induction of A3A is transient in nature with A3A peaking at 72 h after knockdown of PNPase and returning from a 15-fold to a 2-fold induction after an additional 24 h. Thus, the transient induction of A3A by the dysregulation of endogenous nucleic acids could be responsible for some of the proposed episodic bursts of A3A mutagenesis observed in cancer (8, 71).

In the context of both A3A and A3B, episodic mutagenesis by A3A may cause “mutational flares” and A3B may contribute to a continuous “mutational smolder,” which together account for the overall landscape of APOBEC signature mutations in cancer. Because of its potent deaminase activity and capacity to damage the genome, A3A is tightly regulated and only induced in response to infection, inflammation, and other stresses to the cell including mitochondrial dysfunction as shown here. Thus, the transient upregulation of A3A during these conditions could lead to nuclear DNA damage and mutation accumulation. However, A3B, which is nuclear and often expressed at much higher levels in tumors, may result in a continuous but slower accumulation of APOBEC3 signature mutations to the nuclear genome over time. Thus, A3A and A3B can together explain the bulk of the overall APOBEC mutation signature across cancer and the mechanism described here through endogenous dsRNA may be particularly relevant to tumor types with nonviral, nonchronic, or otherwise unclear etiologies.

## Experimental procedures

### Cell culture

MCF10A cells were cultured in Dulbecco's modified Eagle's medium/F12 (Thermo Fisher Scientific #11320033) supplemented with 5% horse serum (Sigma-Aldrich #H1270), 20 ng/ml EGF (Peprotech #AF-100-15), 0.5 μg/ml hydrocortisone (Sigma #H0888), 100 ng/ml cholera toxin (Sigma #C8052), 10 μg/ml insulin (Sigma #91077C), and 1% penicillin/streptomycin (Thermo Fisher Scientific #15140122). HeLa and A549 cells were cultured in Dulbecco's modified Eagle's medium (Thermo Fisher Scientific #SH30022FS) supplemented with 10% fetal bovine serum (Life Technologies #1043702) and 1% penicillin/streptomycin (Thermo Fisher Scientific #15140122). Cells were maintained at 37 °C and 5% CO_2_. MCF10A cells were purchased from Horizon, and A549 cells, HeLa cells, and 293T cell lines were obtained from the American Type Culture Collection (ATCC).

### RNA interference

See Table S1 for all oligonucleotide sequences including siRNA sequences. Duplex siRNAs (IDTDNA) were resuspended at 20 μM in nuclease-free duplex buffer (IDTDNA #11-01-03-01), and cells were treated at a final concentration of 10 nM. siRNAs were reverse transfected using Lipofectamine RNAiMAX (Thermo Fischer Scientific #13778150) in OptiMEM (Thermo Fischer Scientific #31985062). RNAiMAX was used at a ratio of 5 μl to 1 μl of 20 μM siRNA. To transfect plasmid DNA and siRNAs concurrently, TransIT-X2 (Mirus #MIR6000) was used to transfect the plasmid DNA and RNAiMAX was used to transfect the siRNA 24 h later. Transfections of siRNAs were completed in antibiotic-free medium for 72 h before harvesting. siRNA transfection efficiency was assessed using TYE 563 (IDTDNA #51-01-20-19), and knockdown of desired proteins was evaluated *via* immunoblot and RT-qPCR analysis.

### CRISPR knockout cells

MCF10A cells engineered by CRISPR to lack *MAVS* and *STAT2* were described recently (25, 72). See Table S1 for all oligonucleotide sequences including gRNAs. The construct encoding the gRNA was created by cloning the gRNA into the LentiCRISPR1000 (73) plasmid *via* Golden Gate cloning using the Esp3I sites. Virus was created using HEK-293T cells (ATCC) transfected with LentiCRISPR1000 plasmids encoding the gRNA, gag, and VSVG. The gRNA for the *RIG-I* knockouts was 5′- GCGCCTGGACAATGGCACCT-3′, and the gRNA for the *A3A* knockouts was 5′- GAAAAACAACAAGGGCCCAA-3’. MCF10As were then transduced and selected with puromycin (1 μg/ml) (Gold Biotechnology #P-600-500) after 24 h. Surviving cells were single cell cloned in a 96-well plate and grown until 80% confluent. Cells were maintained in 1 μg/ml puromycin for all subsequent passages. Knockout of the target gene was verified by immunoblot and pJet sequencing (Thermo Fisher Scientific #K1231) of the target region. After harvesting genomic DNA from the cells, primers were used to amplify 200 bp surrounding the target sequence on each end (5′-GATGCTCGGTGTGGTAGGAG-3′ and 5′-CCCTGAGTCCTCAGATCCCA-3′ for A3A), which was then cloned into a pJet vector using the CloneJet PCR Kit (Thermo Fisher Scientific #K1231). Ten different plasmids were confirmed using Sanger DNA sequencing (GeneWiz) for each gene.

### Quantitative reverse-transcription PCR

See Table S1 for all oligonucleotide sequences including PCR primers. Total RNA was extracted from cells using the High Pure RNA Isolation Kit (Roche Life Science #11828665001) per the manufacturer’s instructions. The total RNA was transcribed into cDNA in a 20-μl reaction using 50 μM random hexamer primers (5′-NNNNNN-3′) (IDTDNA), 1 mM dNTPs (Millipore Sigma #DNTP-RO), 20 U transcriptor reverse transcriptase (Roche Life Science #3531317001), and 20 U protector RNase inhibitor (Roche Life Science #3335399001). Quantitative PCR was carried out on a LightCycler 480 II (Roche Life Science) in technical triplicate using SsoFast Eva Green Supermix (Bio-Rad #1725200).

### Immunofluorescence microscopy

See Table S2 for information on all primary and secondary antibodies. Cells were fixed in 4% formaldehyde (Thermo Fisher Scientific #28906) for 15 min and permeabilized using PBS containing 0.2% Triton X-100 (Sigma-Aldrich #T8787). Cells were blocked in immunofluorescence microscopy blocking solution (2.8 μM KH_2_PO_4_, 7.2 μM K_2_HPO_4_, 5% goat serum, 5% glycerol, 1% gelatin from cold water fish, 0.04% sodium azide, pH 7.2) with 0.1% Triton X-100 for 1 h at room temperature. Cells were incubated overnight at 4 °C in primary antibody, which was diluted in immunofluorescence blocking buffer. Incubation of cells with fluorophore-conjugated secondary antibody diluted in immunofluorescence blocking buffer was completed for 2 h at room temperature, and nuclei were stained with Hoechst 33342 (1 μg/ml) (Thermo Fisher Scientific #PI62249). Images were collected at 20× magnification (or 10× magnification for Fig. 1*B*) using a Cytation 5 Cell Imaging Multi-Mode Reader (BioTek) or an EVOS FL Cell Imaging System (Thermo Fisher Scientific). For the γ-H2AX images, a Cytation 5 Cell Imaging Multi-Mode Reader was used to image five slices that were 2 μM apart, which were then combined using a maximum intensity projection to create the final image.

### Immunofluorescence microscopy with differential permeabilization

Immunofluorescence microscopy with differential permeabilization was conducted in a manner similar to the immunofluorescence microscopy protocol listed above. However, instead of permeabilizing the cells with PBS containing 0.2% Triton X-100, cells were permeabilized with 0.2% digitonin (Sigma-Aldrich #D141) to ensure permeabilization of all membranes, or 0.02% digitonin to permeabilize only the plasma membranes, or 0% digitonin to permeabilize none of the membranes. Blocking was completed in the same immunofluorescence microscopy blocking solution but without the added 0.1% Triton X-100. The rest of the immunofluorescence microscopy is the same as the immunofluorescence microscopy protocol for the permeabilization of all membranes with Triton X-100.

### Immunoblotting

See Table S2 for information on all primary and secondary antibodies. Cells were lysed in 2.5× RSB (125 mM Tris HCl, 20% glycerol, 7.5% SDS, 5% β-mercaptoethanol, 250 mM DTT, 0.05% Orange G, pH 6.8) and boiled for 10 min. Lysates were run on a 4 to 20% gradient SDS-PAGE gel (Bio-Rad #3450033) and then transferred to a PVDF membrane (Millipore #IPFL00010). Membranes were blocked in 5% milk in PBS for 1 h at room temperature. Primary antibody was diluted in 5% milk in PBS and applied overnight at 4 °C. Blots were then incubated in secondary antibody in 5% milk in PBS supplemented with 0.1% Tween 20 (Thermo Fisher Scientific #BP337-500) and 0.02% SDS (Thermo Fisher Scientific #419530010) for 1 h at room temperature. Blots using the antibody 5210-87-13 (66) for A3A and A3B, as well as antibodies against MAVS, STAT2, ISG15, and SUPV3L1, utilized an HRP-labeled anti-rabbit secondary antibody (Jackson ImmunoResearch #111-035-144), which was visualized using SuperSignal West Femto Maximum Sensitivity Substrate (Thermo Fisher Scientific #PI34095). Blots were imaged on the LI-COR Odyssey Fc imaging system (LI-COR Biosciences).

### Subcellular fractionation

Approximately 10^6^ cells were pelleted for 2 min at 3000 RPM and then washed in 150 μl cold PBS. Cells were pelleted again at 3000 RPM for 2 min. The supernatant was then decanted, and the pellet was resuspended in 90 μl ice-cold 0.1% IGEPAL CA-630 (Sigma-Aldrich #I8896). This resuspension was the whole-cell fraction, and a portion (30 μl) of the resuspension was removed and treated with RSB. The remaining resuspension was pelleted at 3000 RPM for 5 min at 4 °C. The supernatant was the cytosolic fraction, and a portion (30 μl) of the resuspension was removed and treated with RSB. The pellet was then washed in 80 μl ice-cold 0.1% IGEPAL CA-630 (Sigma-Aldrich #I8896) and spun again at 3000 RPM for 5 min. The supernatant was discarded, and the pellet was resuspended in 10 μl HED buffer (25 mM Hepes, 15 mM EDTA, 1 mM DTT, 10% glycerol, pH 7.4). This resuspension was the nuclear fraction and was subsequently treated with RSB.

### Chemicals and inhibitors

Cells were treated with MitoTracker CMXRos (Thermo Fisher Scientific #M7512) for 30 min at a concentration of 500 nM in order to stain the mitochondria prior to fixation. 3p-hpRNA/LyoVec (Invivogen #tlrl-hprnalv) was used at 1 μg/ml for 16 h to stimulate and screen for RIG-I in the control and RIG-I knockout cells. PMA (Sigma-Aldrich #P1585), a known inducer of A3A and A3B (63, 64, 65), was used at 25 ng/ml over 24 h. Doxorubicin (Sigma-Aldrich #D1515) was used as a positive control for DNA damage response by treating cells for 24 h at a concentration of 1 μM.

## Data availability

All relevant data are contained within the main article or supplemental information. Please email rsh@uthscsa.edu with requests for raw data or reagents.

## Supporting information

This article contains supporting information.

## Conflict of interest

The authors declare that they have no conflicts of interest with the contents of this article.
