# Supplementary material for: Mitochondrial double-stranded RNA triggers induction of the antiviral DNA deaminase APOBEC3A and nuclear DNA damage
Source: J Biol Chem. 2023 Jul 19;299(9):105073. doi: 10.1016/j.jbc.2023.105073 (PMC10457583; doi:10.1016/j.jbc.2023.105073)
Supplement: Supporting information [file mmc1.pdf]

Supplementary Information for:

Mitochondrial double-stranded RNA triggers induction of the antiviral DNA deaminase APOBEC3A and nuclear DNA damage

Running title: APOBEC3A upregulation by mitochondrial dsRNA

Chloe Wick[1], Seyed Arad Moghadasi[1], Jordan T. Becker[1], Elisa Fanunza[2,3], Sunwoo Oh[4,5], Elodie Bournique[4,5], Rémi Buisson[4,5], Reuben S. Harris[1,2,6,*]

[1] Department of Biochemistry, Molecular Biology and Biophysics, University of Minnesota, Minneapolis, MN 55455, USA

[2] Department of Biochemistry and Structural Biology, University of Texas Health San Antonio, San Antonio, TX 78229, USA

[3] Department of Life and Environmental Sciences, University of Cagliari, Cittadella Universitaria di Monserrato, Monserrato (Cagliari), SS554, 09042, Italy

[4] Department of Biological Chemistry, School of Medicine, University of California Irvine, Irvine, CA 92697 USA

[5] Center for Epigenetics and Metabolism, Chao Family Comprehensive Cancer Center, University of California Irvine, Irvine, CA 92697, USA.

[6] Howard Hughes Medical Institute, University of Texas Health San Antonio, San Antonio, TX 78229, USA

* Correspondence to Reuben S. Harris, email: rsh@uthscsa.edu

Contents: Supplementary Tables S1-S2, and Supplementary Figures S1-S5

Table S1: Oligonucleotide sequences.

Primer target	Forward	Reverse
TBP	CCCATGACTCCCATGACC	TTTACAACCAAGATTCACTGTGG
APOBEC3A	GAGAAGGGACAAGCACATGG	TGGATCCATCAAGTGTCTGG
APOBEC3B	GACCCTTTGGTCCTTCGAC	GCACAGCCCCAGGAGAAG
APOBEC3C	AGCGCTTCAGAAAAGAGTGG	AAGTTTCGTTCCGATCGTTG
APOBEC3D	ACCCAAACGTCAGTCGAATC	CACATTTCTGCGTGGTTCTC
APOBEC3F	CCGTTTGGACGCAAAGAT	CCAGGTGATCTGGAAACACTT
APOBEC3G	CCGAGGACCCGAAGGTTAC	TCCAACAGTGCTGAAATTCG
APOBEC3H	AGCTGTGGCCAGAAGCAC	CGGAATGTTTCGGCTGTT
PNPT1 (PNPase)	GGAATGGACCTGTTGGGGC	TACCAACTGCTGAATGCCCTG
SUPV3L1	TCACAACCTCTCAAGCCCTG	TGAAGACCAGCTGCCCTTATAG
TDP-43	AAGTTCTTATGGTGCAGGTCAAG	GAGAGAAGAACTCCCGCAGC
DDX58 (RIG-I)	ATGTGCTCCTACAGGTTGTGG	GGGACATTCTCAGCTGTTGC
IFIH1 (MDA5)	TCAAACCCATGACACAGAATGAAC	CTCTCATCAGCTCTGGCTCG
MAVS	CCACACAGCAGGTGCGAC	GGAGCAGATGATAGGCTCGG
STING	TGGGCCTCAAGGGCCTG	ATATACAGCCGCTGGCTCAC
IFNAR1	CAGGAGCGATGAGTCTGTCG	GGACCAATCTGAGCTTTGCG
STAT1	ACCAGAACGAATGAGGGTCC	TTGGAGATCACCACAACGGG
STAT2	GACAAAAGGAGAAAGGAGGTGC	GTTCCAACCCGTGGTCAATG
ISG15	ACAGCCATGGGCTGGGA	GTTCGTCGCATTTGTCCACC
IFI44	GTTGGTAAACGCTGGTGTGG	CCTCCCTTAGATTCCCTATTTGC
DDX60	CAGACGAAGGCCTGAACGAT	AGTCTTGTGTGCCTCTTCCG
MX1	GCGGGATCGTGACCAGATG	CGATGGCATTCTGGGCTTTATT
OAS1	CAAGGTGGTAAAGGGTGGCT	CTCCCCGGCGATTTAACTGA
TNF-α	TGCACTTTGGAGTGATCGGC	CAGCTTGAGGGTTTGCTACAAC
IL-6	GGCACTGGCAGAAAACAACC	TGCATCTAGATTCTTTGCCTTTTTC

siRNA target	Sequence
Ctrl	IDTDNA #51-01-14-04
PNPT1 (PNPase)	GUUUUUACAGAUUACGAGCAUGACA<br>UGUCAUGCUCGUAAUCUGUAAAAACUG
SUPV3L1	ACCUUAAAGAGCCAAGCUAGAAGGA<br>UCCUUCUAGCUUGGCUCUUUAAGGUUC
TDP-43	GAGAGGACUUGAUCAUUAAAGGAAT<br>AUUCCUUUAAUGAUCAAGUCCUCUCCA
DDX58 (RIG-I)	AUAUCAGGUCCUCAAUCUUCAGCTA<br>UAGCUGAAGAUUGAGGACCUGAUAUCA
MDA5	GUCAUCACACCAACAAAGAAGCAGT<br>ACUGCUUCUUUGUUGGUGUGAUGACAU
MAVS	CAGUGCCUUCUAAUGCGCUCACCAA<br>UUGGUGAGCGCAUUAGAAGGCACUGCA
IFNAR1	AAAGCAGCACUACUUACGUCAUGGA<br>UCCAUGACGUAAGUAGUGCUGCUUUAA
STAT1 (Figure 3C)	ACAGAAAGAGCUUGACAGUAAAGTC<br>GACUUUACUGUCAAGCUCUUUCUGUUU

STAT1.1 (Figure S3)	CUUGACAGUAAAGUCAGAAAUGUGA<br>UCACAUUUCUGACUUUACUGUCAAGCU
STAT1.2 (Figure S3)	ACUCAAGAAGAUGUAUUUAAUGCTT<br>AAGCAUUAAAUACAUCUUCUUGAGUAA
STAT2	ACUGAGGAGAAUAUACCUGAAAACC<br>GGUUUUCAGGUAUAUUCUCCUCAGUGA
**CRISPR-Cas9 target**	**gRNA sequence**
DDX58 (RIG-I)	GAAAAACAACAAGGGCCCAA
APOBEC3A	GCGCCTGGACAATGGCACCT

Table S2: Primary and secondary antibodies.

Target	Species and antibody type	Antibody dilution	Company (catalog number)
Tubulin	Mouse monoclonal	1:10000 WB	Sigma-Aldrich (#T6074)
ISG15	Rabbit polyclonal	1:1000 WB	Cell Signaling (#2743)
APOBEC3A APOBEC3B	Rabbit monoclonal	1:1000 WB	5210-87-13 [see reference (66)]
γ-H2AX (pSer139)	Mouse monoclonal	1:300 IF	EMD Millipore (#05-636)
Histone H3	Rabbit polyclonal	1:1000 WB	Abcam (#ab1791)
dsRNA (J2)	Mouse monoclonal	1:200 IF	Jena Bioscience (#RNT-SCI-10010200)
PNPase	Rabbit polyclonal	1:1000 WB	Proteintech (#14487-1-AP)
SUPV3L1	Rabbit polyclonal	1:500 WB	Bethyl Laboratories (#A303-055A)
TDP-43	Rabbit polyclonal	1:1000 WB	Proteintech (#10782-2-AP)
RIG-I	Rabbit monoclonal	1:1000 WB	Cell Signaling (#3743)
MDA5	Rabbit polyclonal	1:1000 WB	Proteintech (#21775-1-AP)
MAVS	Rabbit polyclonal	1:1000 WB	Cell Signaling (#3993)
STAT1	Rabbit polyclonal	1:1000 WB	Cell Signaling (#9172)
STAT2	Rabbit polyclonal	1:1000 WB	Cell Signaling (#4594)
αRabbit HRP	Goat (secondary)	1:5000 WB	Jackson ImmunoResearch Laboratories (#111-035-144)
IR Dye 680 LT αMouse	Goat (secondary)	1:10000 WB	LI-COR (#926-68070)
IR Dye 800CW LT αRabbit	Goat (secondary)	1:5000 WB	LI-COR (#926-32211)
Alexa Fluor 488 αMouse	Goat (secondary)	1:2000 IF	ThermoFisher Scientific (#A28175)

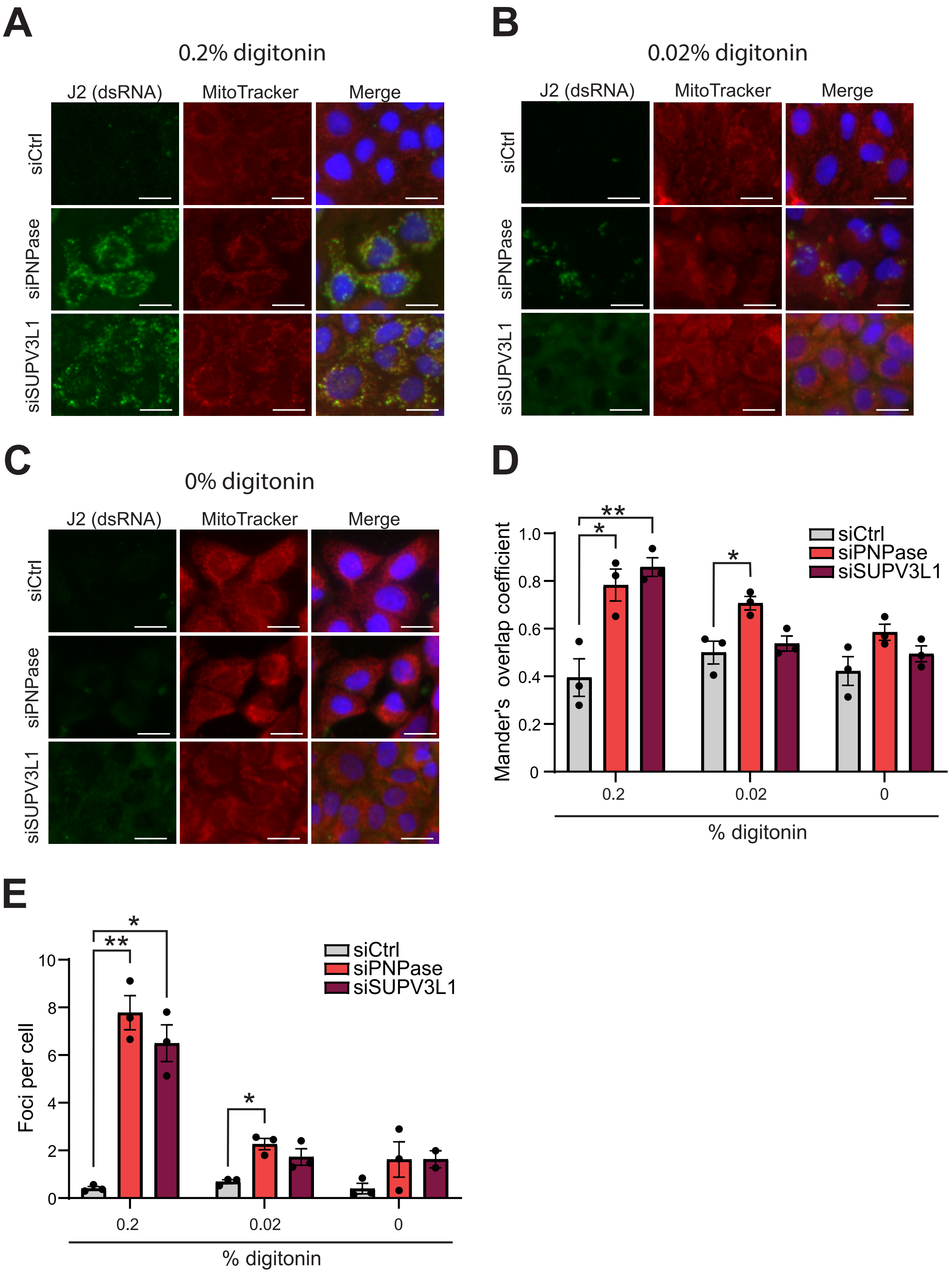
A
0.2% digitonin
J2 (dsRNA)
MitoTracker
Merge
siCtrl
siPNPase
siSUPV3L1
B
0.02% digitonin
J2 (dsRNA)
MitoTracker
Merge
siCtrl
siPNPase
siSUPV3L1
C
0% digitonin
J2 (dsRNA)
MitoTracker
Merge
siCtrl
siPNPase
siSUPV3L1
D
Mander's overlap coefficient
1.0
0.8
0.6
0.4
0.2
0
**
*
*
siCtrl
siPNPase
siSUPV3L1
0.2
0.02
0
% digitonin
E
Foci per cell
10
8
6
4
2
0
*
**
*
siCtrl
siPNPase
siSUPV3L1
0.2
0.02
0
% digitonin

Figure S1. dsRNA present in the cytosol triggers A3A upregulation.

(**A-C**) Immunofluorescence microscopy of MCF10A cells treated with siCtrl, siPNPase, siSUPV3L1, and siTDP-43 for 72h and permeabilized with (A) 0.2% digitonin, (B) 0.02% digitonin, or (C) 0% digitonin (scale bar 10 μm).

(**D**) Mander's overlap coefficient for GFP (dsRNA) overlapping RFP (MitoTracker). Cells were treated with siCtrl, siPNPase, or siSUPV3L1 for 72 hrs. Mean values ± SEM of three independent experiments (*p $\leqslant$ 0.05, **p $\leqslant$ 0.01 by student's t-test and not shown if insignificant).

(**E**) J2-positive dsRNA foci per cell following treatment with siCtrl, siPNPase, or siSUPV3L1 for 72 hrs. Mean values ± SEM of three independent experiments (*p $\leqslant$ 0.05, **p $\leqslant$ 0.01 by student's t-test and not shown if insignificant).

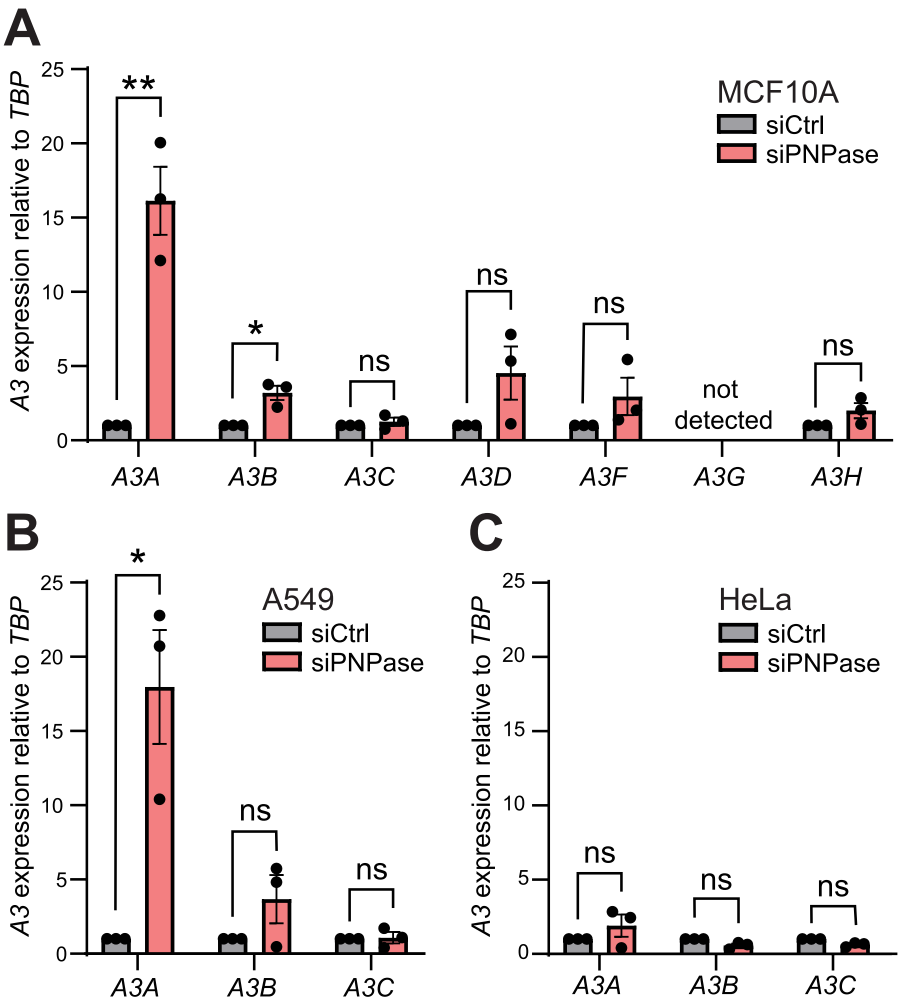

Figure S2. A3A is significantly induced in MCF10A and A549 but not HeLa cells.

(**A-C**) RT-qPCR analysis of the APOBEC3 family of proteins 72h after siPNPase treatment in (A) breast epithelial (MCF10A) cells, (B) lung epithelial (A549) cells, and (C) HeLa cells. Expression refers to mRNA fold change relative to the negative control (which was set to 1) and was normalized to *TBP*. Mean values ± SEM of 3 independent experiments (*p $\leqslant$ 0.05, **p $\leqslant$ 0.01, ***p $\leqslant$ 0.001 by student's t-test; ns, not significant).

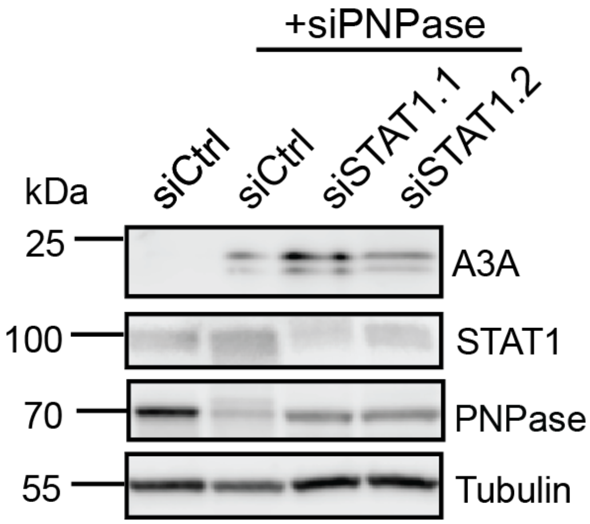

Figure S3. STAT1 depletion has no effect on endogenous A3A levels.

Immunoblot analysis of A3A protein levels in siCtrl or siSTAT1 treated MCF10A cells following siCtrl or siPNPase treatment. The 1.1 and 1.2 siRNAs used here are independent of the siSTAT1 used in Figure 3B-C (sequences in Table S1).

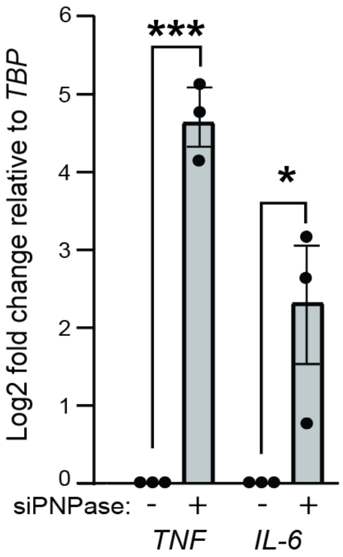

Figure S4. Inflammatory cytokine gene expression in PNPase-depleted cells.

RT-qPCR analysis of tumor necrosis factor alpha (TNF-α) and interleukin 6 (IL-6) mRNA levels 72h after siPNPase treatment of MCF10A cells. Expression refers to mRNA $\log_2$ fold change relative to the negative control (which was set to 0) and was normalized to *TBP*. Mean values ± SEM of 3 independent experiments (*$p \leqslant 0.05$, ***$p \leqslant 0.001$ by student's t-test).

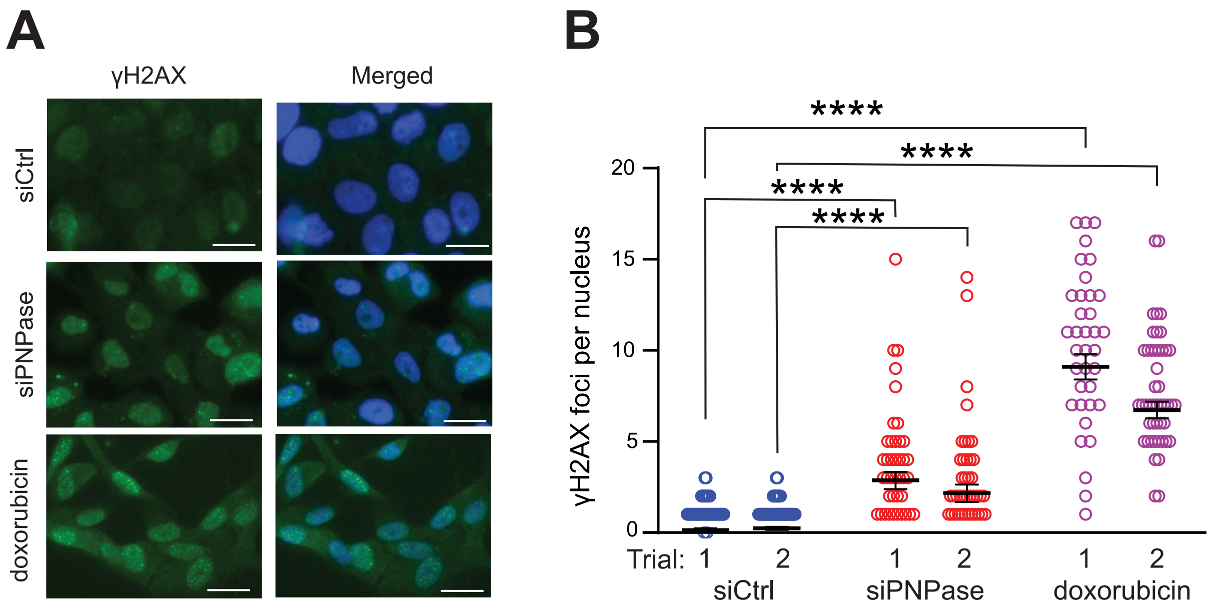

Figure S5. Induction of A3A by PNPase triggers a DNA damage response.

(**A**) Representative immunofluorescence microscopy images of γ-H2AX stained wildtype MCF10A cells treated with siCtrl or siPNPase for 72 hrs or 1 μM doxorubicin for 24 hrs. Images were taken using an EVOS FL Imaging System at 20x magnification (10 μm scale bar).

(**B**) Quantification of the number of the number of γ-H2AX foci per nucleus in the experiment shown in panel A, as well as another independent experiment. Mean values ± SEM (2 sets of independent biological replicates with n>50 cells each; ****p $\leqslant$0.0001 by student's t-test).
